# Conducting co-creation for public health in low and middle-income countries: a systematic review and key informant perspectives on implementation barriers and facilitators

**DOI:** 10.1186/s12992-024-01014-2

**Published:** 2024-01-17

**Authors:** Giuliana Raffaella Longworth, Oritseweyinmi Erikowa-Orighoye, Ebuka Miracle Anieto, Danielle Marie Agnello, Jorge Raul Zapata-Restrepo, Caroline Masquillier, Maria Giné-Garriga

**Affiliations:** 1 Faculty of Psychology, Education and Sport Sciences, Universitat Ramon Llul, Blanquerna, Barcelona, Spain; 2 Newcastle University, Newcastle upon Tyne, UK; 3 School of Health and Life Sciences, Glasgow Caledonian University, Glasgow, UK; 4 School of Health and Sports Sciences, University of Suffolk, Ipswich, UK; 5 Family Medicine and Population Health’– FAMPOP, Faculty of Medical and Health Sciences & ‘Centre for Family, Population and Health, Faculty of Social sciences, University of Antwerp, Belgium, Belgium; 6 Faculty of Health Sciences, Universitat Ramon Llull, Blanquerna, Barcelona, Spain

**Keywords:** Co-creation, LMICs, Implementation, Review, Facilitators, Barriers

## Abstract

**Background:**

There has been an increase in the use of co-creation for public health because of its claimed potential to increase an intervention’s impact, spark change and co-create knowledge. Still, little is reported on its use in low-and-middle-income countries (LMICs). This study offers a comprehensive overview of co-creation used in public-health-related interventions, including the interventions’ characteristics, and reported implementation barriers and facilitators.

**Methods:**

We conducted a systematic review within the Scopus and PubMed databases, a Google Scholar search, and a manual search in two grey literature databases related to participatory research. We further conducted eight interviews with first authors, randomly selected from included studies, to validate and enrich the systematic review findings.

**Results:**

Through our review, we identified a total of twenty-two studies conducted in twenty-four LMIC countries. Majority of the interventions were designed directly within the LMIC setting. Aside from one, all studies were published between 2019 and 2023. Most studies adopted a co-creation approach, while some reported on the use of co-production, co-design, and co-development, combined either with community-based participatory research, participatory action research or citizen science. Among the most reported implementation barriers, we found the challenge of understanding and accounting for systemic conditions, such as the individual’s socioeconomic status and concerns related to funding constraints and length of the process. Several studies described the importance of creating a safe space, relying on local resources, and involving existing stakeholders in the process from the development stage throughout, including future and potential implementors. High relevance was also given to the performance of a contextual and/or needs assessment and careful tailoring of strategies and methods.

**Conclusion:**

This study provides a systematic overview of previously conducted studies and of reported implementation barriers and facilitators. It identifies implementation barriers such as the setting’s systemic conditions, the socioeconomic status and funding constrains along with facilitators such as the involvement of local stakeholders and future implementors throughout, the tailoring of the process to the population of interest and participants and contextual assessment. By incorporating review and interview findings, the study aims to provide practical insights and recommendations for guiding future research and policy.

**Supplementary Information:**

The online version contains supplementary material available at 10.1186/s12992-024-01014-2.

## Background

People living in socio-economically vulnerable circumstances in LMICs might stand at the crossroad of different vulnerabilities which may reinforce each other, creating a complex system of challenging living circumstances. Vulnerability might result from developmental problems, personal incapacities, disadvantaged social status, the inadequacy of interpersonal networks and supports, degraded neighbourhoods and environments [1–3]. As argued previously, these social determinants of health are closely linked to unequal distribution of wealth, power and resources and should be viewed in the perspective of global socio-economic disparities [4].

This economic and developmental disparity is evident in the context of public health interventions developed in the donor country, i.e. countries which provide aid to a developing country. This relationship, characterized by a donor and receiving country, highlights the existing inequalities between a donor country, which is typically more economically advanced and a received country, which is faced with critical issues such as critical issues like poverty, overburdened health systems and limited resources for education [5].

Those type of interventions have been criticized, not only by their reinforcement of existing neoliberal practices and lack of acknowledgement for existing power relationships [4] but also for their lack of effectiveness and difficulty in addressing real-world concerns critical to the social, cultural and political context within the LMIC setting [6]. Several authors have, in fact, encouraged the use of co-creation as a way to respond to LMICs’ specific needs and settings when developing interventions and/or research agendas aimed at promoting socioeconomic and health development [7–11].

Co-creation is advocated as a collaborative approach to developing solutions, such as interventions aimed at enhancing public health, ensuring that these solutions align with the needs and preferences of stakeholders and the target population. It involves engaging with diverse stakeholders at all project stages, from determining and defining the problem through to the final stages of a project [12]. It involves the engagement of stakeholders from all nodes of the quadruple helix (academia, industry, government, and users) to co-create effective and sustainable solutions [13] and has been shown to be a promising approach to increase the impact of health interventions, especially in vulnerable populations [14, 15].

The approach has been regarded as a means, specifically in LMIC settings, to account for existing hierarchies, increase the value for partners involved [9] and intervention’s impact by addressing issues that are felt as pressing to the community and/or the targeted population [10, 16]. Co-creation has been considered as a way to involve representatives of the target population and stakeholders as part of the process seen as a way to understand the system and related influencing factors [15, 17]. Accounting for health systems-level facilitators and barriers, independently of the implementing organization, has been regarded crucial for contexts which might rely on fragile health systems [18].

In 2020, a comprehensive global assessment of reviews on co-design carried out by Slattery et al. documented the reported advantages linked with the use of co-design. The primary benefits included were mostly associated with positive emotional outcomes, increased self-management, applicability and acceptability of the research questions and positive impacts on the researcher(s) involved [19]. The study also described the challenges of such an approach, including time and financial resources constraints, tensions between researchers and end-users in decision-making, and the balance between scientific rigour and end-user preferences [20].

A literature review study by Singh et al. [19] examined co-design research for healthcare in LMICs [19]. The study identified challenges, such as limited budget and time to evaluate the intervention outcomes; low literacy of people living in socioeconomic vulnerable circumstances participating in the co-design process, challenges in addressing existing power dynamics; poor governance; balancing of diversity in participation, existing gender-related norms and traditional values [19].

This study aims to further Singh et al.’s literature review [19] in two ways. Firstly, it aims to enhance the methodology by performing a systematic search, as opposed to relying solely on the authors’ prior knowledge of relevant studies as done by Singh and colleagues. Secondly, by conducting anonymous interviews, the study aims to validate the included studies’ retrieved data and to extract nuanced insights into the challenges and facilitators experienced during the co-creation process.

Building on the work presented by the studies mentioned above, this review aims to understand whether co-creation has been and, if so, how it has been used in the context of LMICs for interventions addressing public health issues. All studies included in the review comply with a definition of co-creation intended as a collaborative approach of creative problem solving engaging diverse stakeholders at all project stages, from determining and/or defining the problem through to the final stages of a project [12].

In the context of LMICs for people living in socio-economically vulnerable circumstances, the review aims to meet the following research objectives:

- To identify public health interventions that have adopted a co-creation approach (according to the previous definition) in LMICs with people living in vulnerable socioeconomic circumstances;
- To explore the interventions’ main characteristics, such as geographical distribution, the approach adopted, the origin of the intervention and stakeholders’ type of involvement;
- To identify the authors’ perceived implementation barriers and facilitators.

## Methods

### Systematic review

The review was conducted following the Preferred Reporting Items for Systematic Review and Meta-analyses (PRISMA) guidelines [21]. To identify relevant case studies, we have systematically reviewed interventions using co-creation in the context of LMICs for people living in socio-economically vulnerable circumstances within the PubMed and Scopus databases and grey literature in two open-source databases. We then randomized studies to conduct eight semi-structured interviews with the first authors to further explore their perceived implementation barriers and facilitators.

### Search strategy

A search strategy was developed and applied to the PubMed and Scopus databases. The search combined terms related to co-creation, co-research, co-investigation, co-design, co-production and co-development together with public health-related terms and a list of low and middle-income countries (Additional File 1). An overview of the differences between the co-approaches can be found elsewhere [12], as this goes beyond the scope of this study. No time or language limits were imposed on the search.

Despite the two research methodologies of community-based participatory research (CBPR) and co-creation being close in the extent to which end-users are engaged and scope, CBPR-based projects have already been reviewed in several papers [22–24]. Thus, this review does not include community-based participatory research (CBPR) studies. However, some studies reported on the use of the CBPR in combination with the approaches included in the search strategy. The search terms are reported in Table 1 and full details available in Additional File 1.

**Table 1:** Overview of search strategy

Co-creation	Public health block	LMIC country bloc
“co-creat*” OR “co-research*” OR “co-investigat*” OR “co-develop*” OR “co-invent*” OR “co-produc*” OR “co-design*”	“Public health” OR “health promotion “ OR “community health” OR “epidemiolog*” OR “environmental health” OR “health education” OR “prevent*”	“Afghanistan” OR “Albania” OR ·Algeria” OR “Angola” OR “Argentina” Or “Armenia” OR “Belarus” (full list of countries available in Additional File 1).

We further scanned for grey literature by conducting the following searches: (a) a search within Google Scholar, using the first 200 records as recommended by Haddaway et al. [25], and (b) by conducting a manual search including policy briefs and reports by research institutions in the following databases: Patient-Centered Outcomes Research Institute (PCORI)and Participedia. Details of the search strategy for each database is included in Additional File 1.

### Eligibility criteria

The study design according to the PICO framework was the following:

#### Population

Targeted people living in LMICs according to the Organisation for Economic Co-operation and Development’s (OECD) list of 2022–2023 [26] and in socio-economically vulnerable circumstances. The latter was described as a ‘neighbourhood socioeconomic disadvantage’ (including but not limited to disadvantaged communities, poverty, neighbourhood/area status); or any definition of ‘individually measured disadvantage’ (including but not limited to low income, entitlement to medical or other state benefits, unemployment, low educational status and social class) [27].

#### Interventions/comparators

Public health interventions adopting a co-creation approach, described as a collaborative approach of creative problem solving engaging diverse stakeholders at all project stages, from determining and/or defining the problem through to the final stages of a project [12]. Studies might not have explicitly used co-creation as a term, but should comply with the definition above. Public health was defined as all organised measures (whether public or private) to prevent disease, promote health, and prolong life among the population as a whole [28].

### Outcomes

Study design: Empirical studies, i.e., studies reporting results/lessons learnt of a conducted process.

### Interviews

To ensure that we collected the authors’ key perspectives on the barriers and facilitators of conducting co-creation in low and middle-income countries and validate our data extraction’s relevance and understanding, we performed eight semi-structured with the selected studies’ first authors.

We randomly selected studies and reached out to the first authors for the interviews via email. We followed-up with invited interviewees once and, if no reply was received after one week, we moved on to the next interviewee identified by the randomization. Out of the eight contacted initially, six responded while the following two interviewee were next on the randomization list.

Interviews were anonymized, and all participants were asked to share information around age, gender and describe their occupation and years of experience with co-creation (Additional File 3). Interview followed a semi-structure format and questions related to implementation barriers and facilitators felt as hindering or supporting the intervention and on general recommendations for future interventions (Additional File 4). All interviews were audiotaped with the informed consent of the participants, and verbatim transcription conducted for each interview.

A thematic analysis guided the interpretation of the interviews’ findings, following the six stages outlined by Braun and Clarke [29], and by doing so, we have undertaken the following steps; (1) GL and DA familiarized with the data and wrote familiarization notes; (2) GL and DA developed a systematic data coding for four of the interviewees; (3) GL and DA independent generating initial themes from coded and collated data; (4) finally met to develop and reviewing themes; and to (5) refining, defining and naming themes; and (6) applied the thematic framework to the remaining interviews. As advocated by Braun and Clarke [29] we meant for our coding to be open, with no use of coding framework. The themes reported in the results section emerged as the result of the data coding and iterative theme development [29].

### Data extraction

All titles, abstracts, and full texts were screened by two independent reviewers against the inclusion criteria. Rayan software was used for both title and abstract and full-text screening. From the included papers, we extracted information related to details of the studies, including the aim, context and public health issue addressed, together with details around approaches used, type and role of the facilitators, implementation barriers and facilitators reported in the paper.

Data extraction from papers was performed according to a pre-set list of study characteristics (Additional File 2) and conducted by two authors independently. If any, doubts were solved until reaching a consensus (OO, GL). The list of included studies, together with key intervention characteristics, are summarised and reported in Table 2.

### Data analysis

Data related to the implementation barriers and facilitators were then categorized and summarised in Figs. 2 and 3 into components deriving from the updated Consolidated Framework for Implementation Research (CFIR) [30] and further elaborated upon in the Results section.

The CFIR framework has been previously used and considered valuable to contextualize study findings in the context of LMICs [18]. As part of the review and as an additional consideration for using the framework, authors highlighted the importance of accounting for health system-level facilitators and barriers, which might be independent of the individual but affecting the interventions’ frame and context.

Incorporating insights from the CFIR framework review findings [18], we adopted a definition for the Outer setting, which emphasized the relevance and influence of systemic factors rather than on the individual i.e. the various contextual factors, components, and elements that collectively shape and influence the functioning, behaviour, and outcomes of a given system [31].

We described the Outer Setting as the setting in which the Inner Setting exists, including the local environments, such as the hospital system, school district, state, but also as the wider socioeconomic environment in which the intervention is taking place, including systemic-level implementation facilitators and enablers. The inner setting is the setting in which the innovation is implemented, e.g., hospital, school, city; and the intervention implementation setting is the activities and strategies used to implement the intervention [30].

We categorized results into the following CFIR components, namely:

• Implementation barriers:
- Outer setting: Local conditions, Policies & Laws
- Inner setting: Access to Knowledge & Information, Relational connection
- Intervention: Engaging
• Implementation facilitators:
- Intervention implementation: Engaging, Teaming, Tailoring Strategies, Adapting, Assessing Needs, and Evaluating.

## Results

**Fig. 1:**
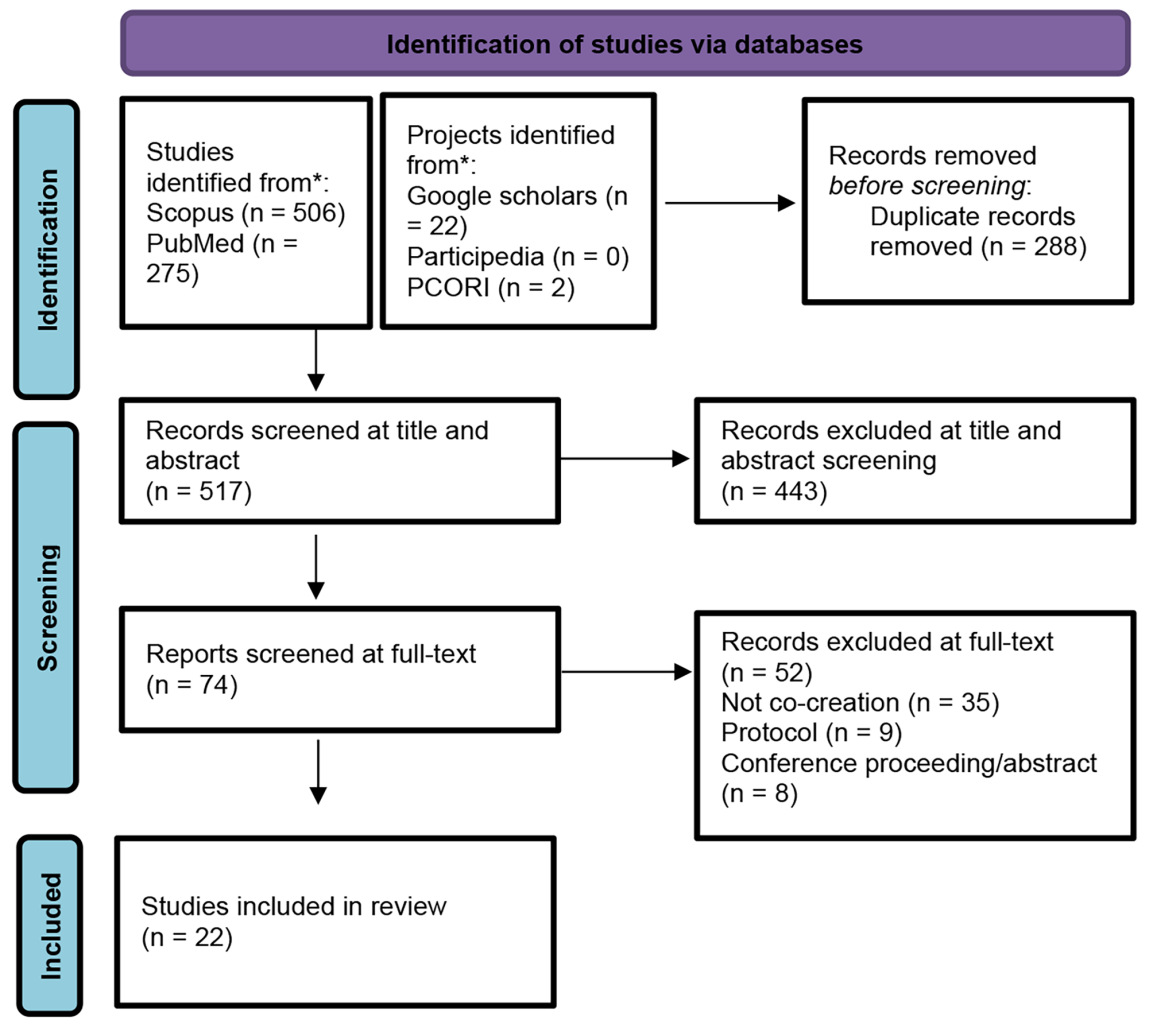
PRISMA Flow

The screening process of included studies is reported below in the PRISMA Flow below (Fig. 1). Twenty-two studies were included.

We summarised the selected studies’ details in Table 2, including information about authors, year of publication, public health issues and challenges addressed, as well as details about the co-creators participating in the intervention and the study’s aim.

**Table 2:** Studies included

Authors	Year	Country	Public health issue	Challenge addressed	Stakeholders involved	Aim
Chenais et al. [32]	2023	Uganda	African swine fever	African swine fever (ASF) spread along the smallholder value chain.	Community members, including all value chain stakeholders, including pig farmers, traders, slaughter slab operators, butchers, pork vendors and pork joint owners	To investigate the capacity of participatory action at the community level for improved biosecurity in the smallholder pig value chain in Northern Uganda.
Maureithi et al. [33]	2023	Malawi	HIV prevention	Need to develop digital health applications fitting contextual resource constraints.	HIV patients, healthcare workers, clinical and evaluation team	To design a text-based system of tailored reminders and messages.
Robbins et al. [34]	2022	Haiti and Zimbabwe	Pre-eclampsia	High maternal mortality ratios in Haiti and Zimbabwe.	Community members, health professionals, representatives from non-governmental organizations (NGOs) and representatives from the ministries of health	To develop culturally relevant, context-specific educational resources through interdisciplinary collaboration and community engagement.
Ilozumba et al. [35]	2022	Uganda	Cancer prevention	Low use of interactive voice response (IVR) technology for cancer information provision.	People living with cancer, health workers, professional roles (doctors, oncologists, nurses, etc.) and policy makers (e.g., from the Ministry of Health and other regulatory bodies)	To develop a program theory for a co-created IVR system for cancer awareness in Uganda.
Willetts et al. [36]	2022	Indonesia	Climate change and sanitation	Impact of climate-related hazards and impact on sanitation systems.	City governments	To co-produce a research process with local governments.
Almeida et al. [37]	2022	Brazil, Paraguay, and Argentina	Alcohol, tobacco, and cannabis consumption.	Regions of the study report a high percentage of criminal activity and economic disadvantage.	Researchers and representatives of the target population	To co-develop activities as part of an intersectoral intervention tackling alcohol, tobacco, and cannabis consumption among teenagers.
Corburn et al. [38]	2022	Kenya	Climate change, WASH, flooding.	Urban informal settlements or slums are among the most vulnerable to climate-related health risks.	Researchers, community members and representatives of the target population	To co-create an innovative climate justice plan.
Tahlil et al. [39]	2022	Nigeria	HIV prevention	Low uptake of HIV testing services and poor rates of linkage to care.	Researchers and community members	To develop HIV self-testing (HIVST) strategies for potential implementation in their local communities.
Banerjee et al. [40]	2022	India	Vaccine campaigns	Population resistance to vaccine campaigns.	Community members, stakeholders and faith leaders.	To co-design interventions to address vaccine hesitancy.
Nwaozuru et al. [41]	2021	Nigeria	HIV	Low uptake of HIV testing services and poor rates of linkage to care.	Young people, health professionals, activists, and entrepreneurs.	To develop a deep understanding of their needs and to maximize user satisfaction with HIV services.
Hartmann et al. [42]	2021	South Africa	HIV prevention	Low uptake of HIV testing services and poor rates of linkage to care.	Youth	To develop long-acting biomedical HIV prevention programs.
Shahmanesh et al. [43]	2021	South Africa	HIV prevention	Low uptake of HIV testing services and poor rates of linkage to care.	Youth	To develop a peer-led biosocial intervention for HIV prevention.
Anwar et al. [44]	2021	Egypt	HIV	Fishermen have limited access to health services and may have various health literacy-related difficulties that may lead to poor health outcomes.	Fishermen and health workers	To co-design health literacy interventions with the aim of improving health and equity outcomes.
Boateng et al. [45]	2021	Ghana	Health literacy in management of malaria	Struggle to eradicate malaria in Ghana and high rates of positivity to disease in children under five years old.	Target population and relevant stakeholders.	To develop a health literacy intervention for Ghana caregivers concerning managing malaria in children under five years.
Yadav et al. [46]	2021	Nepal	Multi-morbid chronic obstructive pulmonary disease (COPD)	Nepal has the highest prevalence of COPD in South Asia.	Persons with COPD and their family members; healthcare providers and other stakeholders, including community leaders, representatives from government.	To develop an integrated model of care for people with multi-morbid COPD in rural Nepal.
Echaubard et al. [47]	2020	Cambodia	Dengue and dengue control	Dengue is the most rapidly spreading mosquito-borne viral disease in the world and is strongly related to urban expansion worldwide, particularly in tropical regions.	Teachers, school directors and Ministry of Education representatives and students.	To operationalize and sustain community-led dengue control operations.
Brakema et al. [48]	2020	Kyrgyzstan and Vietnam	Chronic respiratory diseases	A high number of death rates to chronic respiratory diseases in LMICs	Researchers and Community members	To study the feasibility, acceptability, and effectiveness of translating an awareness programme targeting risks to Cardiorespiratory diseases to Kyrgyzstan and Vietnam.
Santina et al. [49]	2020	Lebanon	Childhood obesity	In Lebanon, 31.4% of children aged 5–19 years were reported to be overweight or obese.	Community members	To increase the moderate to vigorous physical activity (PA) level at school among Lebanese children aged 10–12.
Lazo-Porras et al. [50]	2020	Peru	Chronic diseases at the primary healthcare level	85% of premature deaths due to non- communicable diseases (NCDs) occur in low- and middle-income countries (LMICs).	Community members, health workers, and policymakers	To develop interventions aimed at improving diagnosis and/or management of chronic diseases at the primary healthcare level.
Crosby et al. [51]	2020	India, Sierra Leone	Hand-hygiene	The potential of hand-hygiene strategies to control disease transmission.	Research team and stakeholders, including teachers and collaborators.	To adapt culturally relevant resources for hand-hygiene awareness.
Draper et al. [52]	2019	South Africa	Obesity prevention	Increase obesity levels.	Community members	To co-develop a healthy lifestyle programme for low-income, black South Africans through churches.
Person et al. [53]	2016	Tanzania	Urogenital schistosomiasis control	Lack of effectiveness of previous interventions.	Target population and relevant stakeholders.	To develop and implement interventions to track local infections.

Most interventions were designed directly in the LMIC setting [32, 34, 36–38, 40–43, 45–47, 50, 53, 54], while two interventions were initially designed and implemented in another LMIC and then transferred and adapted to the study’s LMIC setting [35, 48], one transition occurring within the same continent, moving from Ghana and Nigeria to Uganda [35], while the other involving a transfer from Vietnam and Kyrgyzstan to Uganda [48]. Three interventions were initially designed in a HIC setting and transferred and adapted to an LMIC setting [33, 44, 51].

The majority of interventions were conceived and designed within LMIC settings, with two being transferred between different LMIC contexts, and an additional three originating in a high-income country (HIC) before being adapted to LMIC settings. The studies in which the original intervention was designed in the HIC all highlighted the relevance of conducting a contextual adaptation phase. One study undertook the identification of local needs and a co-design intervention phase [44] while the other two studies conducted focus groups and interviews with local stakeholders for the adaptation of the intervention’s materials [33, 51].

We applied no time limitation on the search strategies, but all included papers were published between 2019 and 2023 and one study was published in 2016 [53].

**Fig. 2:**
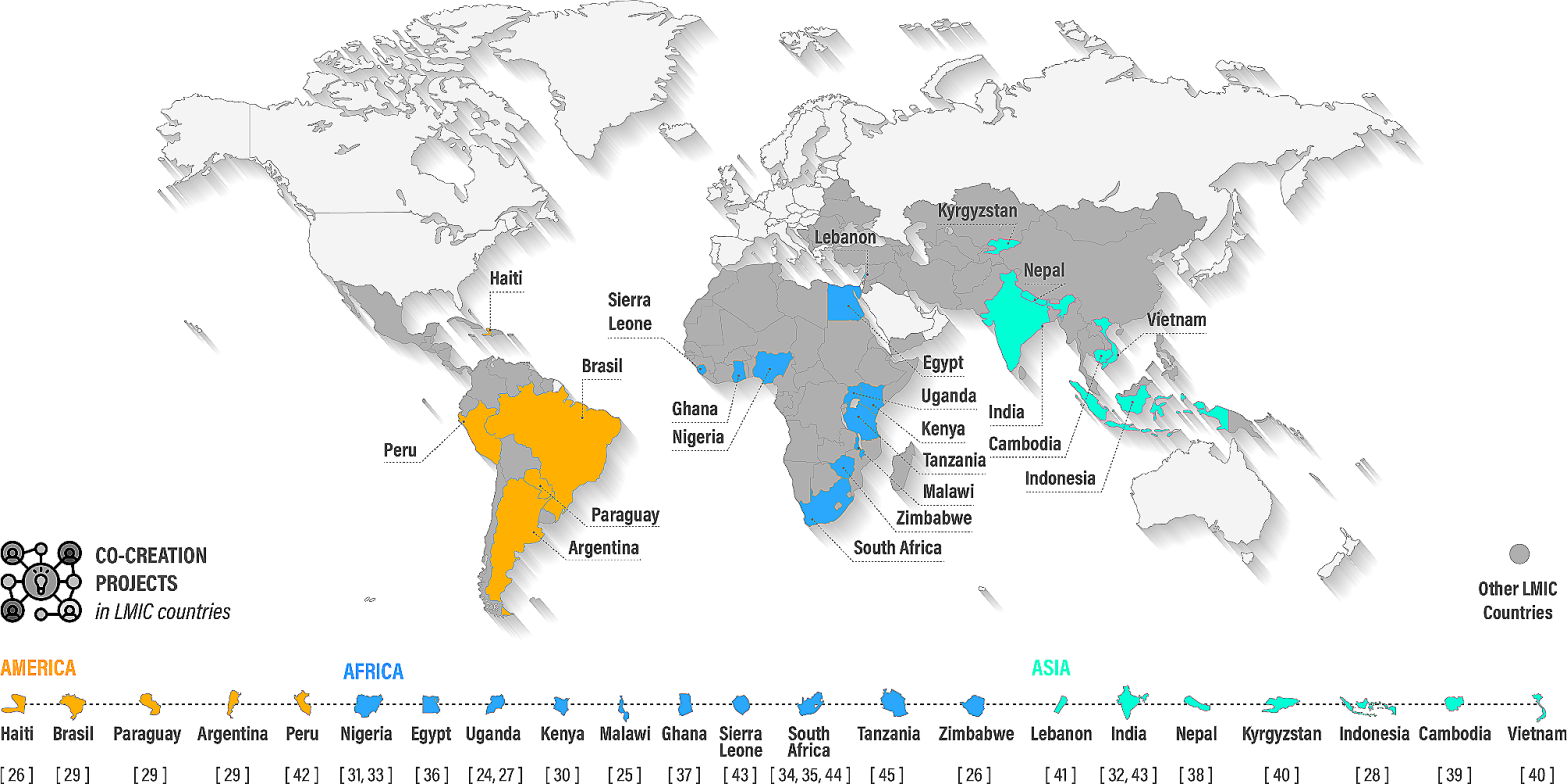
Geographical distribution of co-creation projects in LMIC countries, including references

We represented the geographical distribution of studies in Fig. 2.

### Overview of participatory approaches adopted

Studies adopted one or a combination of the following approaches: co-creation, co-production, co-design, co-production and co-development. Studies, in some cases, complemented these approaches with CBPR, participatory action research, citizen science, and human-centred design. The majority of studies reported adopting solely a co-creation approach [33, 33–35, 39, 41, 45, 48, 50, 51], while others used co-creation in combination with CBPR [43, 47] or with co-design [38, 46, 53] or with co-production and participatory action research [42]. Co-creation was also combined with co-design and CBPR [40]. Some adopted a co-production approach [36] while others used the approach of co-design [48], co-development [37, 52]. Others utilized human-centred design [53] or citizen science as their approach when co-creating [38]. One of the studies adopted a co-design approach combined with CBPR [44].

Despite having involved end-users at different moments of the intervention, all the authors engaged with representatives from people living in socio-economically vulnerable circumstances when developing the intervention’s outputs.

Several authors have been engaging with representatives of the people living in socio-economically vulnerable circumstances to conduct needs and contextual assessment before the intervention’s design [34, 35, 38, 45, 47, 53, 55] while some have also involved representatives from the target population also when co-designing the intervention [34, 36, 38, 41–43, 49, 50, 52, 53].

#### Implementation barriers

**Fig. 3:**
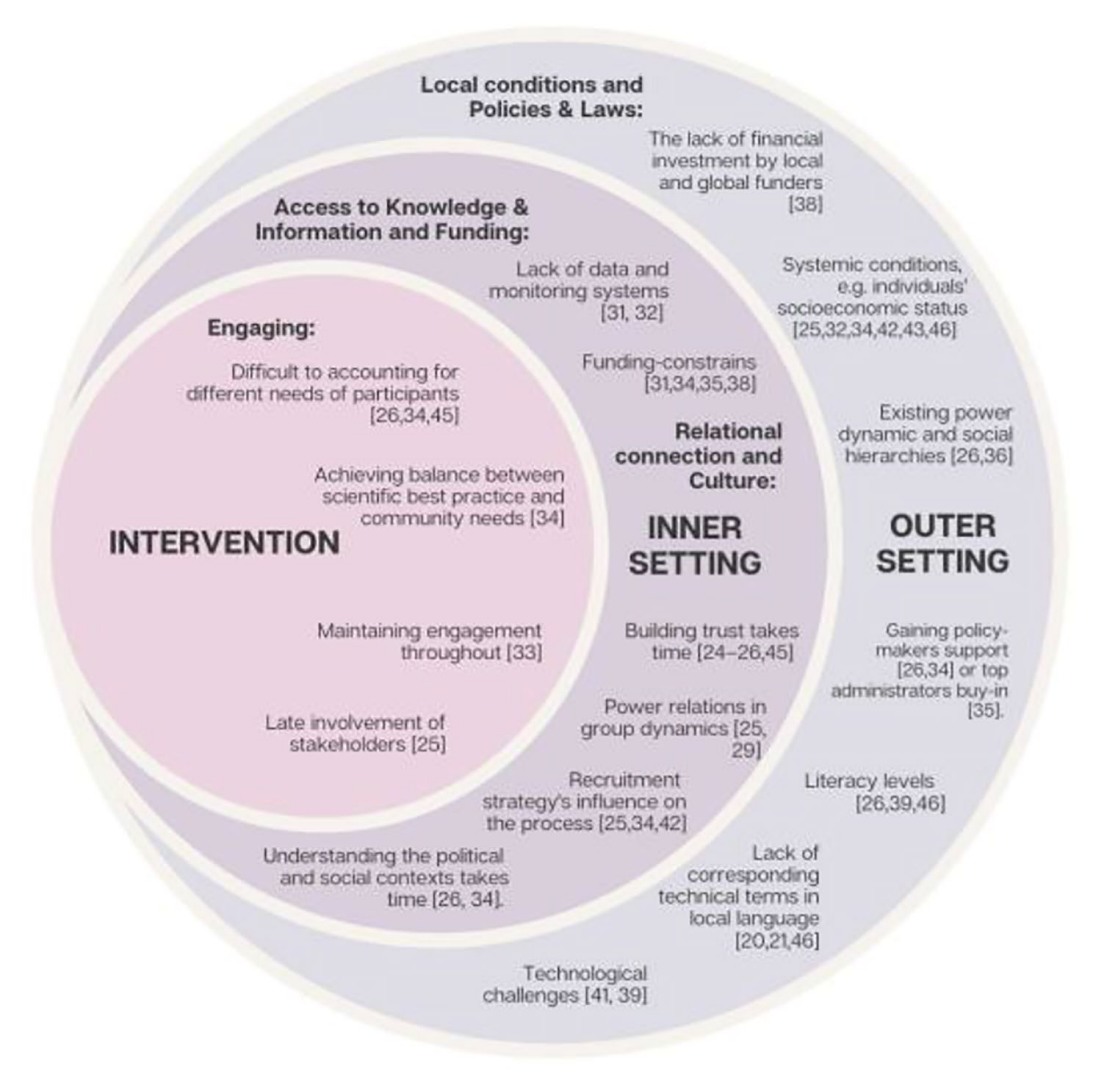
Implementation barriers when co-creating in LMIC settings

To group and present implementation barriers and facilitators’ results, we used the CFIR components [30] as described in the Methods section and represented in Fig. 3.

### Outer setting

Outer setting is described as the setting in which the Inner setting exists, including the local environments, such as the hospital system, school district, state, but also as the wider socioeconomic environment in which the intervention is taking place, including systemic-level implementation facilitators and enablers [18].

#### Local conditions and policies & laws

The lack of financial investment in flexible processes by local and global funders.

have been reported to cause difficulty in the implementation of co-creation projects [38].

Systemic conditions related to the participants’ context, such as the individual’s socioeconomic status, including factors related to household composition, parental education and difficulty to absent from work for participation in the sessions, are mentioned, by several authors, as an important influential condition which must be taken into account when developing and implementing co-creation [32, 37, 44, 50–52]. Several studies [34, 35, 38, 45, 47, 53, 55], in fact, recommend conducting a needs assessment and investigating contextual factors influencing the context and issue, as described in the ‘Needs assessment’ paragraph in the implementation facilitators section of this manuscript.

The impact of existing entrenched power dynamics and social hierarchies within the settings and between stakeholders has been said to influence the project dynamics [34, 46]. Some authors experienced difficulties gaining policymakers’ support [47, 50] or top administrators’ buy-in [45].

Several studies reported participants’ varying literacy levels and formal education impact on the process [34, 35, 52]. A lack of shared language and equivalents of technical terms in the local language was expressed as a challenge in conveying an understanding of the subject matter [20, 21, 40], together with difficulty in accounting for all the variety of languages spoken within the same local setting [34]. Some participants experienced technological challenges [33] and, more specifically, difficulties in accessing and charging mobile phones [35] or related to a weak network system [33].

### Inner setting

The inner setting is the setting in which the innovation is implemented, e.g., a hospital, school, city.

#### Access to knowledge & information and funding

Lack of data on subject matters related to the monitoring of facilities, specifically on disaster hazards [36] but also access to an extensive list of socioeconomic indicators [37], has been proven difficult. The need to better share monitoring information and data across agencies was highlighted [36].

Some reported the process as time and effort intensive [41, 50] stressing this might represent a more significant challenge when set in resource-limited settings because of the limited availability and capacity of targeted participants [41]. Time-intensity concerns the amount of time asked of individuals involved and the amount needed in order to build relationships with stakeholders and to develop the set of facilitation skills needed by all researchers [50]. Several authors identified funding constraints as a key barrier [36, 38, 45, 50].

#### Relational connection and culture

Fostering trust and developing shared objectives is crucial but makes the process lengthy [32, 34, 49, 54] and attention should be placed on existing power relations and group dynamics [32], including the power relation existing between the community members and the research team [42].

Ways in which the recruitment and involvement of stakeholders took place might impact the process itself [32, 44, 50]. For instance, a study reported the backlash encountered by participants that questioned why other community members had not been invited to the intervention and highlighted the importance of carefully planning and thinking about recruitment while leaving the process open for participation [32].

Several authors in the literature have highlighted the considerable challenge of effectively engaging with participants deeply entrenched in complex sociocultural contexts. These contexts encompass a wide array of interwoven social, cultural, historical, and environmental factors that significantly shape individuals’ beliefs, behaviours, and perceptions, making it essential for researchers and practitioners to navigate and comprehend these intricacies when establishing meaningful engagement [32, 50].

Several authors mentioned the challenge of engaging with participants deeply rooted in sociocultural contexts, which implies a process that might have to include time and specific activities to comprehensively grasp the local socioeconomic system and factors related to the context’s needs and challenges [16, 25].

### Intervention implementation setting

The intervention implementation setting is intended to include barriers that relate to the activities and strategies used to implement the intervention [30].

#### Engaging

Authors reported on the challenge of accounting for the different range of needs and motivations of different participants [34, 49, 50] and achieving a balance between scientific best practices and community needs [50]. Maintaining engagement throughout proved challenging [33], and delays in the involvement of relevant stakeholders in the process were thought to have caused a negative impact on the engagement [32].

All the implementation barriers, as described above, are summarized in Fig. 3.

#### Implementation facilitators

**Fig. 4:**
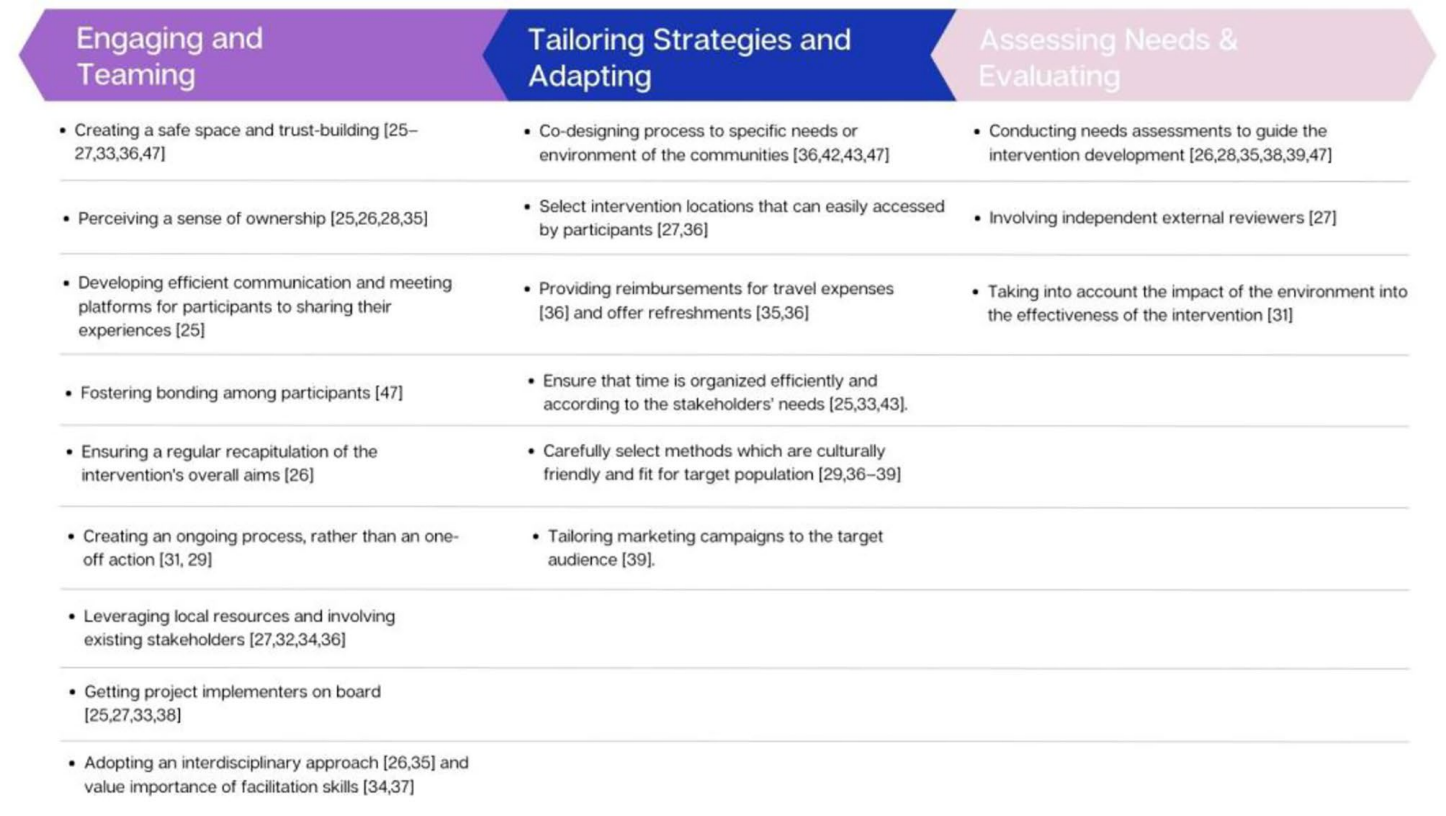
Implementation facilitators when co-creating in LMIC settings

Implementation facilitators reported in included studies concerned the intervention implementation setting, meaning all reported facilitators concerned the setting of the intervention’s implementation. Facilitators were grouped into the CFIR components of Engaging, Teaming, Tailoring Strategies, Adapting, Assessing Needs, and Evaluating. Figure 4 represents the themes identified.

### Intervention implementation setting

The intervention implementation setting is intended to include barriers that relate to the activities and strategies used to implement the intervention [30].

#### Engaging and teaming

Creating a safe space for participants and trust-building was seen as key and highly influential to the intervention’s positive outcomes [32, 34, 40, 41, 46, 55]. Perceiving a sense of ownership, feeling listened and believing that the intervention is responding to the communities’ needs have been reported as elements that may positively affect the intervention by making it ownable, actionable and sustainable [32, 34, 45, 47].

Meaningful engagement was reported as positively influencing the process and outcome [41], allowing participants to gradually acquire a stronger sense of ownership and voice their own needs and insights [34, 47]. To ensure this, some authors suggested developing efficient communication and meeting platforms that allow participants to feel safe when sharing their experiences [32], paying particular attention to fostering bonding among participants [55] and ensuring a regular recapitulation of the intervention’s overall aims [34].

Some authors reported the importance of allowing for an ongoing process rather than a one-off action [36] as this would enable participants to feel part of the process and assimilate learnings and for their perspectives to be fully comprehended and integrated into the intervention’s results [42].

Leveraging local resources and involving existing stakeholders with existing relationships with the target population and deeper local knowledge of the context have been considered crucial to the success of the intervention [37, 41, 46, 50]. Some studies mainly highlight the importance of getting future and potential intervention implementers on board and close to participants, increasing, this way, the chance of successfully delivering or maintaining the solution [32, 38, 40, 41]. Nurturing and maintaining these stakeholder partnerships has been seen as a crucial aspect of the process [38].

To enable meaningful engagement, some recommend adopting an interdisciplinary approach involving people from different disciplines and with diverse backgrounds and experiences as much as a variety of stakeholders [34, 50]. Furthermore, some studies report good facilitation skills as crucial to the process but potentially challenging because of differences among participants in motivation, personalities, socioeconomic factors, and affecting the capacity of involvement and power dynamics within the group [50, 53].

#### Tailoring strategies and adapting

Several authors reported the need for co-designing processes, which suit the specific needs or environment of the communities [44, 46, 51, 55]. To do so, some authors recommended selecting locations for co-creation sessions that can easily be accessed by participants [41, 46], providing reimbursements for travel expenses [46] and offering refreshments [45, 46] and ensuring that time is organized efficiently and according to the stakeholders’ needs [32, 40, 51].

Furthermore, studies highlighted the importance of carefully selecting methods which are culturally friendly and chosen according to the target population. Choosing creative and tailored methods during the sessions allowed participants who may have been reluctant and/or unused to share and present ideas to engage in the process [35, 38, 42, 46, 53]. A study also reported on the importance of tailoring marketing campaigns to the target audience [35].

#### Assessing needs & evaluating

To tailor interventions, conducting needs assessments to guide the intervention development has been considered important to ensure the project’s success [34, 35, 38, 45, 47, 55]. To gather information about the contextual factors influencing the public health issue, before the intervention’s development, some authors have conducted semi-structured interviews and focus group discussions with community members [34] while others have also included participant observations, information conversations with community and stakeholders [47].

In terms of evaluation, some have appreciated the involvement of independent external reviewers [41] and the importance of considering the environmental impact on the intervention’s effectiveness [36].

All the implementation facilitators were grouped into the CFIR components of Engaging, Teaming, Tailoring Strategies, Adapting, Assessing Needs, and Evaluating and summarized in Fig. 4.

### Thematic analysis of interviews

We conducted eight semi-structured interviews with the study’s first authors to validate and expand further on implementation barriers and facilitators experienced throughout the intervention, as well as provide some lessons learned and tips for future researchers taking on co-creation projects in LMICs. We have reported on key themes and relevant quotes below. Recommendations provided by interviewees are reported in Table 3.

### Socioeconomic conditions

Echoing the insights from the included articles, several interviewees shared difficulties linked to the socioeconomic circumstances of the participants engaged in the process, highlighting an impact on the intervention. Interviewee 6 shares that “you have to be more creative in a low resource setting and tailoring, I guess, that’s important even more in the settings, as you say, there’s different levels of literacy”, expressing the importance of tailoring your process to be able to overcome challenges. Interviewee 2 stressed the impact of the country’s structural challenges on the implementation of the project by stating “you have differences within the villages and by difference, I mean in experiences and poverty levels and knowledge and then you have also differences between villages. “.

Interviewee 8 mentioned, in this respect, the challenge, while co-creating, of dealing with “competing interests and priorities”. They say “unemployment is extremely high [and] we were engaging with young people of varying ages, but those who might not be in school and have finished school and ultimately want to earn an income. And you’re asking them to contribute their time and effort. And often don’t have the capability to pay them as employees”. They further say, “We always had reimbursements for participation and transport and food. But, you know, I don’t know if that’s always fair, especially in this type of context when people are, you know, they’re also looking maybe to make connections in the hopes that it might lead to some sort of employment” (interviewee 8).

Interviewee 5 provides the example of participants who had to share phones with family and/or have a prepaid number, which made communication’s maintenance difficult, sharing that “a lot of times they’re sharing phones with people and their family don’t necessarily have their own phones or might have like a prepaid number and that changes so maintaining communication and contact is difficult”. Interviewee 4 shares the challenge of engaging, through the intervention, with professionals that are already overburdened with emergencies and issues.

### Funding

Multiple interviewees emphasized the challenge of funding, a concern that was also documented in several of the included studies. One interviewee brought up the challenges faced while seeking approval from their University Ethics Committee. The interviewee found it difficult to present their intervention plan since the scope and approach depended on community input, leading to uncertainty. They say “so when I was applying to the ethics committee in [name of country, omitted for anonymity purposes], they asked me like, what do you need to do? You are going to have to identify a goal. I was like, oh, I don’t know what I’m doing. What are your outputs going to be? Because I know the community, they are going to choose my output. It took around three to four months. And they always said me like, what you are going to develop? You need to mention. I said like, look, in co-design, we don’t know what we are going to develop. That’s the beauty. It’s the community-chosen approach” (Interviewee 7).

The same interviewee stresses the limitations that might be caused by the fact that “donor agencies don’t have clear funding structures to support this innovative process in the LMICs”. The interviewee says “whether the European Union or the National Institute for Health and Care Research (NIHR), or any other agency, donor agencies should support studies that are created and led by the local communities together with local investigators”. Similar considerations are expressed by Interviewee 2, who believes that “donor agencies, they don’t have clear funding structures, how to support this innovative process in the LMICs”.

Interviewee 4 appreciates the intervention’s funding organism as they provided them with flexibility but also mentions that “in a lot of other funding mechanisms, you have even less ability [and] if it takes a bit longer to engage a group of young people, for example, and that’s conflicting with your other priorities and timelines, then it can definitely all fall by the wayside”. Interviewee 1 also believed the funders’ willingness to provide resources without a strict intervention plan helped to conduct the intervention in line with the community’s needs and feedback.

### Recruitment and meaningful engagement

Resonating with what was mentioned in included papers, interviewees emphasized the significance of establishing trust with the community members. interviewee 7 emphasized that “you need to explain to that community, you need to understand the community dynamics, you need to understand how the community functions, you need to understand the cultural way of living, you need to fit into that! You don’t ask the people to fit into your way of thinking”. To achieve a meaningful engagement, the interviewer invested a substantial amount of time immersing themselves in the community before the intervention commenced, to ensure a solid foundation of trust was built.

Transparency and honesty emerged as recurring themes among several authors. They shared that, in their view, it was fundamental to “share the knowledge and action plan step by step, which made the community comfortable. I didn’t have to worry about how to recruit the participants because the community took the lead” (interviewee 5). By openly communicating about available resources, one author cited showing the community the received funds on a laptop by saying, “This is the money I have; I can show you. So that’s what I showed them, and I told them this is what we have to do the work” (interviewee 5).

In this particular scenario, community members placed their trust in the researcher, leading them to actively participate in workshops, data collection, and advocating for the project’s continuation with local decision-makers. Interviewee 5 recounted that, when faced with funding uncertainty, they approached the community and honestly communicated the situation, stating, “I don’t have the funding now, and I don’t know what to do”. In response, the community suggested approaching the local government, and they promptly took action, approaching the local government to seek support and ensure the project’s continuity.

Lastly, the facilitator’s proficiency in the community’s languages proved to be valuable, enabling smooth negotiations, trust-building, and eliminating language barriers. Having fun throughout the process also played a significant role. In one of the occasions, “the community prepared the food, [and] served it to the politicians. They really enjoyed it. It was like a fun party for them” (interviewee 5).

### Partnering and power dynamics

Despite the initial challenges in gaining the trust of local leaders, partnering was deemed essential for the project’s success, as also highlighted in included papers as a crucial implementation facilitator. “I picked some of the social leaders who were influential leaders and who could advocate for me, which helped to reach out to some of the outliners in the communities,” says interviewee 8. To build trust and address concerns, the interviewee found it crucial to present the stakeholders with tangible examples of the outputs that would directly benefit the community.

Interviewee 4 emphasized the advantages of involving local stakeholders stating “we have some partners that helped us to set up the dates, to organize the meetings, to invite all the different community members that we would like to have or involve in our co-creation meetings”.

At the data analysis stage, collaboration and solid partnership proved also to be crucial. Interviewee 5 highlights “this [the local stakeholders’ involvement] helped us accommodating their [the community’s] needs, times, wishes, etc.” and “this [the local stakeholders’ involvement was] something that was really valuable and powerful was their role in helping to interpret the results”.

Bringing together the diverse array of stakeholders meant also having to deal with potential power imbalances, as expressed by Interviewee 6, 4 and 5. Interviewee 6 mentions “I think that you need to prepare and you need to know that you have that power and how you try to not use it and how you like try to work with your team of facilitators to avoid that. Interviewee 4 shares that it was important to try “minimize those [power imbalances] as much as possible by treating the collaboration as more of a relationship and partnership as opposed to, you know, leading partner and implementing partner, trying to offer an approach which is more of like, hey, we’re research partners on this”. Similarly, interviewee 6 shares that “the motivation [behind the project] was to was mostly just to be able to collaborate with people from the community instead of kind of treating people in the community as implementing partners”.

To address power imbalances, interviewee 5 had “initially separate negotiations with each stakeholder”. Then “brought all of them at the one space, where they feel comfortable. What I did in the very beginning is to like make them understand what is power, what is people and how important we are.” Interviewee 5 shares that it was important to try “minimize those [power imbalances] as much as possible by treating the collaboration as more of a relationship and partnership as opposed to, you know, leading partner and implementing partner, trying to offer an approach which is more of like, hey, we’re research partners on this”. Interviewee 5 had “ initially separate negotiations with each stakeholder”. Then “brought all of them at the one space, where they feel comfortable. What I did in the very beginning is to like make them understand what is power, what is people and how important we are”.

### Tailoring

In the same way included studies highlighted the importance of tailoring, multiple interviewees mentioned it as a crucial facilitator. Interviewee 6 highlighted how it aided to explain complex theories and approaches in a simple and relatable language.

Interviewee 4 says “the motivation came from having the intervention tailored to what the needs of their community and what they thought work and could be effective.” The same interviewee highlighted that it was a case of “collaborating with people from the community instead of treating them solely as implementing partners”.

Adapting tools and methods also proved to be an advantageous aspect, as mentioned by Interviewees 4 and 1. Both stressed the significance of selecting methods which are appropriate for the target groups to ensure sustained engagement throughout the process. Interviewee 1 additionally encouraged creativity when incorporating activities into workshops to enhance participant interest and involvement.

### Time and facilitation

Several authors identified time as a significant barrier to successful implementation, reinforcing the findings from included studies. Interviewee 6 specifically highlighted the time-consuming process of gaining the community’s trust while also juggling multiple roles as you “collect the data, come home, do the analysis, write the paper. So, in that, I felt like you need to work around the clock”. Interviewee 1 also described the considerable time investment required to set up dates, organize meetings, and invite various community members to the co-creation sessions.

Interviewee 4 mentions “a tension with the priority of wanting to be really participatory and engaged, and then also meeting research study timelines that you need to maintain for your funder.” Moreover, Interviewee 2 expresses that time pressures from publishing deadlines added to the strain, as hosting institutions needed materials for advocacy purposes to support the continuation of such activities.

For Interviewee 1, time constraints were also evident during the formative stages of the project. In this respect, they say that they “felt [like] there wasn’t enough time to digest the information from the formative stages of the project. It takes a lot of time to gather data, analyze it and then contact all the relevant stakeholders.” In this context, effective facilitation played a crucial role in managing and optimizing time during the project’s execution.

### Recommendations

Interviewees were asked to provide a few recommendations for practitioners willing to use co-creation for public health interventions in LMIC settings (Table 3).

**Table 3:** List of recommendations by interviewees

**Tailoring strategies and adapting**
Be very much culturally sensitive and friendly (Interviewee 6).
Researchers should not have their own mission. They need to go with the people’s mission (Interviewee 6).
You always need to prepare like nothing of what you decide is going to work. Be flexible (Interviewee 6).
Meet people where they are. Tailor your approach and methods to the setting in which you are working. Even within the same country, from community to community, an approach is going to need to vary (Interviewee 3)
**Engaging and teaming**
Make sure that the process is truly co-created and not a tokenistic way of saying, Oh, we got feedback from those people (Interviewee 8).
Be honest with your community about what you will have at the end of this process. (Interviewee 2).
Be creative in the activities that you plan (Interviewee 1).
Create a multidisciplinary team (Interviewee 1).
Be as inclusive and representative as possible to enhance the reach and involve people that might not be as enthusiast as others (Interviewee 8).
**Assessing needs and evaluating**
Try to ensure that those interventions are sustainable. For instance, make sure, from the beginning, that the intervention is evaluated in terms of feasibility (Interviewee 8).
Really understand the data before starting with your thorough analysis (Interviewee 7).
Communicate and talk to as many people as you can about the project to understand the context and intervention (Interviewee 5).
It is hard to address the tensions that exist with more traditional research approach and wanting to quantify the value of participation. You don’t necessarily always have to quantify its value to argue for its importance (Interviewee 4).

## Discussion

Through this systematic review, conducted with no time restrictions on the search strategy, apart from a study published in 2016, the review identifies public health interventions conducted in LMICs with people living in socio-economically vulnerable circumstances which have adopted a co-creation approach. All included studies have been published between the years 2019 and 2023. This recent and increasing number of publications reporting on the use of co-creation in LMIC settings testimonies the approach’s vast spreading in the field of international development. It responds, we believe, to a need for addressing local needs and issues, and developing and delivering interventions which fit appropriately local contexts and systems.

Studies included varying co-creation approaches and stakeholders involved. However, studies were included if the engagement was perceived to be done in a meaningful way and not by mere consultation, and if they were complying, according to reviewers, to an approach which included active stakeholder engagement from problem exploration to solution creation, implementation and/or evaluation [12].

All included studies were redeemed as engaging meaningfully with people living in socioeconomic vulnerable circumstances in the development of the intervention’s outputs.

Some studies also engaged representatives of the target population when conducting needs and contextual assessment before the intervention’s design, and some also involved representatives from the target population when co-designing the intervention.

Previous reviews, such as the review conducted by Slattery et al. [20] about co-design in health, found similar results in the reporting of impactful benefits related to the co-design approach, including the tailoring of the research topic, question and materials, as well as allowing for an increase in applicability and acceptability. Acknowledgment for power dynamics, time and financial constraints, as echoed by this study’s results, are also highlighted as main challenges by Slattery et al. [20] and by Singh et al. study on co-design in LMIC countries [19]. In the same way this review reports on socioeconomic conditions as potential challenges to the approach, Singh et al. [19] highlighted compliances related to the health system that may impact the intervention.

To the Singh’s et al. [19] review, this study adds several considerations. We further report challenges related to recruitment and to the balance to be found between ensuring representation and an optimal sampling. Evaluation becomes trickier when dealing with a more flexible process and with a samples of co-creators that may be smaller or not be so easy to strictly control for. Several sampling techniques have been suggested to guide the co-creation process [56] but further investigation is needed on how these may function in practice or work in lower resource settings.

Adding to existing reviews, this study’s results highlight the reported interest in, and importance given to a contextual assessment carried out prior to the intervention as a way to understand and comprehend deeply the socioeconomic and political contexts in which it is operating. By conducting thorough needs and contextual assessments, interventions can strive to align with local needs and challenges [57, 58]. For interventions and solutions to be contextually relevant and effective, they must seek not only meaningful engagement but also a profound comprehension of the specific setting in which solutions are to be implemented [57].

Mansaray et al. [59] for instance highlight that partnering with local communities across the project sites enabled them to get a comprehensive overview of what is already happening on the ground and in the area and to reach out to trusted community members for their advice, support and local knowledge.

Studies identified through this review all recognize the value of involving relevant stakeholders in using co-creation processes to shape and implement interventions. The high degree of participation that characterizes co-creation not only fosters a dynamic and inclusive environment for knowledge creation, translation and exchange, but also ensures that the outcomes truly resonate with the realities and aspirations of the communities and interventions’ target population involved. Studies report [41, 60] that partnering with local NGOs contributed to the solutions’ uptake, recruitment and coordination of the intervention.

Insights from the interviews also further reveal a concern around the lack of funding and financial mechanisms from donor organization that may allow for a meaningful co-creation process. In this respect, further research is encouraged towards funding mechanisms and characteristics that may allow for meaningful co-creation to happen.

By advocating for active collaboration between and with diverse stakeholders, including local communities, NGOs, policymakers, and academics, co-creation seems to enable a shift from traditional top-down approaches and a more nuanced understanding of the complex challenges faced by people living in vulnerable socioeconomic circumstances in LMICs, while fostering innovative and context-specific solutions. As this emerging body of work expands, it signifies a positive stride towards fostering meaningful collaborations and nurturing sustainable, context-sensitive solutions for LMICs.

### Limitations

The study might limitation lies in the potential for bias introduced by the moving on to the next interviewee if a response was not received within a week. This practice could result in a sample that skews towards authors who can respond quickly, potentially differing from those who do not. Furthermore, the study’s supplementary data reveals that most interviewees were relatively young and had not engaged in co-creation research for an extended period. This implies that authors who responded promptly might be at an earlier career stage, while more senior researchers, burdened with larger workloads, may have been less responsive.

The focus of the study primarily centres on the viewpoint of involved researchers, providing insights into what they perceive as effective in co-creation. However, the study lacks exploration of community-level co-creators’ perspectives and how participants experienced barriers and facilitators in the co-creation process. Therefore, it should be noted that reported perceptions stem from the lens of the lead researchers but not necessarily from a community perspective.

Additional we would like to note that, if the studies did not explicitly specify whether the target population was in vulnerable circumstances, the authors exercised their discretion to determine inclusion or exclusion based on their own judgment and consensus. Similarly, reviewers included studies if they perceived them as complying with the definition of co-creation also relies on the reviewers’ own perceptions.

## Conclusion

Results reported in this study should be able to guide researchers and practitioners willing to adopt a co-creation approach in low and middle-income settings by offering an overview of previously conducted research and by presenting potential implementation barriers and facilitators and general recommendations.

Among the most reported implementation barriers, we found the challenge and relevance of understanding and accounting for systemic conditions, such as the individual’s socioeconomic status and elements related to funding constraints and the length of the process. At a systemic level, several authors highlighted the difficulty of engaging with a population that might be already overburdened and struggling with socioeconomic-related conditions, such as lack of financial stability, working burdens, lower literacy levels and coexistence of several pressing issues. Implementation barriers highlighted by both the systematic review and interviewees’ findings reported difficulties in dealing with time and strict funding rules and timelines.

Leveraging local resources by involving and partnering with local stakeholders has been seen as vital by many included studies. In this respect, local stakeholders helped understand the context and encouraged the engagement of co-creators. High relevance is also given to the performance of a contextual and/or needs assessment and careful tailoring of strategies and methods.

Studies identified through this review all recognize the value of involving relevant stakeholders in using co-creation processes to shape and implement interventions. The high degree of participation that characterizes co-creation not only fosters a dynamic and inclusive environment for knowledge creation, translation and exchange, but also ensures that the outcomes truly resonate with the realities and aspirations of the communities and interventions’ target population involved.

Particularly noteworthy are two barriers reported by interviewees, including the challenges posed by the University demands in terms of ethics approval procedures and the pressure that academic researchers face to publish within relatively short time frames. The reported challenges prompt a critical reflection on the modalities that research institution should consider adopting to better support co-creation practices. The latter might include considering a revision of existing structures and expectations surrounding ethics approval processes and academic publication timelines. Arguably, adapting ethics procedures and creating the space for more flexible publication schedules could contribute to fostering a research environment which allows for meaningful co-creation to take place.

In the trajectory of advancing knowledge and practices, we believe future research should look into the funding, structural and operational mechanisms needed to enable such co-creation processes.

### Electronic supplementary material

Below is the link to the electronic supplementary material.

**Supplementary Material 1:** Search strategy

**Supplementary Material 2:** Data Extraction Template

**Supplementary Material 3:** Interviewee details

**Supplementary Material 4:** Guiding interview questions

## Data Availability

All data sources are referenced and in the public domain.

## Abbreviations

LMIC: Low-and-middle-income country
HIC: High-income country
CBPR: Community-based participatory research
CFIR: Consolidated Framework for Implementation Research (CFIR)
