# Supplementary material for: Conducting co-creation for public health in low and middle-income countries: a systematic review and key informant perspectives on implementation barriers and facilitators
Source: Global Health. 2024 Jan 17;20:9. doi: 10.1186/s12992-024-01014-2 (PMC10795424; doi:10.1186/s12992-024-01014-2)
Supplement: Supplementary file 1 — Supplementary Material 1: Search strategy [file 12992_2024_1014_MOESM1_ESM.docx]

|  | Interviewee 1 | Interviewee 2 | Interviewee 3 | Interviewee 4 | Interviewee 5 | Interviewee 6 | Interviewee 7 | Interviewee 8 |
| --- | --- | --- | --- | --- | --- | --- | --- | --- |
| Age | 32 | 33 | 48 | 40 | 35 | 30 | 33 | 31 |
| Gender | Female | Male | Associate professor | Female | Female | Female | Female | Female |
| Occupation | Graduate student | Implementation science researcher | Researcher | Public health researcher | Researcher | Researcher | Researcher | Assistant Professor |
| Years of experience with co-creation | 5 | 8 | 3 | 5 | 5 | 3 | 6 | 5 |
