# Supplementary material for: Conducting co-creation for public health in low and middle-income countries: a systematic review and key informant perspectives on implementation barriers and facilitators
Source: Global Health. 2024 Jan 17;20:9. doi: 10.1186/s12992-024-01014-2 (PMC10795424; doi:10.1186/s12992-024-01014-2)
Supplement: Supplementary file 2 — Supplementary Material 2: Data Extraction Template [file 12992_2024_1014_MOESM2_ESM.docx]

*Intros*

Could you please state the justification and main aim of your study?

*(here we want to know why they decided to conduct the study, what was the motivation, and the main aims, any follow-ups they might have undertaken).*

What implementation barriers did you face throughout the project? Could you recall a barrier that could not be overcome? Would you point out a specific barrier?

*Describe what we mean by implementation barriers and other challenges. We do not want only a “list” of barriers, we want them to tell us the most relevant barriers and whether they could overcome them. If they did, how did they overcome them?*

Can you identify any implementation facilitator that you feel helped the participatory process?

*Describe what we mean by implementation facilitators - focus might be on implementation facilitators during the participatory process.*

What would your key recommendations to professionals using co-creation in LMIC settings be?

*Any additions*
