# Supplementary material for: Conducting co-creation for public health in low and middle-income countries: a systematic review and key informant perspectives on implementation barriers and facilitators
Source: Global Health. 2024 Jan 17;20:9. doi: 10.1186/s12992-024-01014-2 (PMC10795424; doi:10.1186/s12992-024-01014-2)
Supplement: Supplementary file 3 — Supplementary Material 3: Interviewee details [file 12992_2024_1014_MOESM3_ESM.docx]

**PubMed**

| **Co-creation block #1** | TITLE/ABSTRACT (co-creat% OR co-research% OR co-investigat% OR co-develop% OR co-invent% OR co-produc% OR co-design) |
| --- | --- |
| **Public health block #2:** | TITLE/ABSTRACT (public health OR health promotion OR community health OR epidemiolog% OR environmental health OR health education OR prevent%) |
| **LMIC country block #3** | TITLE/ABSTRACT (Afghanistan OR Albania OR Algeria OR Angola OR Argentina OR Armenia OR Azerbaijan OR Bangladesh OR Belarus OR Belize OR Benin OR Bhutan OR Bolivia OR Bosnia and Herzegovina OR Botswana OR Brazil OR Burkina Faso OR Burundi OR Cabo Verde OR Cambodia OR Cameroon OR Central African Republic OR Chad OR China (People's Republic of) OR Colombia OR Comoros OR Democratic Republic of Congo OR Congo OR Costa Rica OR Côte d'Ivoire OR Cuba OR Djibouti OR Dominica OR Dominican Republic OR Ecuador OR Egypt OR El Salvador OR Equatorial Guinea OR Eritrea OR Eswatini OR Ethiopia OR Fiji OR Gabon OR Gambia OR Georgia OR Ghana OR Grenada OR Guatemala OR Guinea OR Guinea-Bissau OR Guyana OR Haiti OR Honduras OR India OR Indonesia OR Iran OR Iraq OR Jamaica OR Jordan OR Kazakhstan OR Kenya OR Kiribati OR Democratic People's Republic of Korea OR Kosovo OR Kyrgyzstan OR Lao People's Democratic Republic OR Lebanon OR Lesotho OR Liberia OR Libya OR North Macedonia OR Madagascar OR Malawi OR Malaysia OR Maldives OR Mali OR Marshall Islands OR Mauritania OR Mauritius OR Mexico OR Micronesia OR Moldova OR Mongolia OR Montenegro OR Montserrat OR Morocco OR Mozambique OR Myanmar OR Namibia OR Nauru OR Nepal OR Nicaragua OR Niger OR Nigeria OR Niue OR Pakistan OR Panama OR Papua New Guinea OR Paraguay OR Peru OR Philippines OR Rwanda OR Saint Helena OR Samoa OR São Tomé and Príncipe OR Senegal OR Serbia OR Sierra Leone OR Solomon Islands OR Somalia OR South Africa OR South Sudan OR Sri Lanka OR Saint Lucia OR Saint Vincent and the Grenadines OR Sudan OR Suriname OR Syrian Arab Republic OR Tajikistan OR Tanzania OR Thailand OR Timor-Leste OR Togo OR Tokelau OR Tonga OR Tunisia OR Turkey OR Turkmenistan OR Tuvalu OR Uganda OR Ukraine OR Uzbekistan OR Vanuatu OR Venezuela OR Vietnam OR Wallis and Futuna OR West Bank and Gaza Strip OR Yemen OR Zambia OR Zimbabwe); OR ((low income OR middle income) OR ((low AND middle[Title/Abstract]) AND countr*)) |
| **#3** | **#1 and #2 AND #3** |

**Scopus**

| **Co-creation block #1** | (((TITLE-ABS-KEY (co-creat% OR co-research% OR co-investigat% OR co-develop% OR co-invent% OR co-produc% OR co-design)) |
| --- | --- |
| **Public health block #2:** | (TITLE-ABS-KEY (public health OR health promotion OR community health OR epidemiolog% OR environmental health OR health education OR prevent%)) |
| **LMIC country block #3** | (TITLE-ABS-KEY (Afghanistan OR Albania OR Algeria OR Angola OR Argentina OR Armenia OR Azerbaijan OR Bangladesh OR Belarus OR Belize OR Benin OR Bhutan OR Bolivia OR Bosnia and Herzegovina OR Botswana OR Brazil OR Burkina Faso OR Burundi OR Cabo Verde OR Cambodia OR Cameroon OR Central African Republic OR Chad OR "China (People's Republic of)" OR Colombia OR Comoros OR "Democratic Republic of Congo" OR Congo OR "Costa Rica" OR "Côte d'Ivoire" OR Cuba OR Djibouti OR Dominica OR "Dominican Republic" OR Ecuador OR Egypt OR "El Salvador" OR "Equatorial Guinea" OR Eritrea OR Eswatini OR Ethiopia OR Fiji OR Gabon OR Gambia OR Georgia OR Ghana OR Grenada OR Guatemala OR Guinea OR "Guinea-Bissau" OR Guyana OR Haiti OR Honduras OR India OR Indonesia OR Iran OR Iraq OR Jamaica OR Jordan OR Kazakhstan OR Kenya OR Kiribati OR "Democratic People's Republic of Korea" OR Kosovo OR Kyrgyzstan OR "Lao People's Democratic Republic" OR Lebanon OR Lesotho OR Liberia OR Libya OR "North Macedonia" OR Madagascar OR Malawi OR Malaysia OR Maldives OR Mali OR "Marshall Islands" OR Mauritania OR Mauritius OR Mexico OR Micronesia OR Moldova OR Mongolia OR Montenegro OR Montserrat OR Morocco OR Mozambique OR Myanmar OR Namibia OR Nauru OR Nepal OR Nicaragua OR Niger OR Nigeria OR Niue OR Pakistan OR Panama OR "Papua New Guinea" OR Paraguay OR Peru OR Philippines OR Rwanda OR "Saint Helena" OR Samoa OR "São Tomé and Príncipe" OR Senegal OR Serbia OR "Sierra Leone" OR "Solomon Islands" OR Somalia OR "South Africa" OR "South Sudan" OR "Sri Lanka" OR "Saint Lucia" OR "Saint Vincent and the Grenadines" OR Sudan OR Suriname OR "Syrian Arab Republic" OR Tajikistan OR Tanzania OR Thailand OR "Timor-Leste" OR Togo OR Tokelau OR Tonga OR Tunisia OR Turkey OR Turkmenistan OR Tuvalu OR Uganda OR Ukraine OR Uzbekistan OR Vanuatu OR Venezuela OR Vietnam OR "Wallis and Futuna" OR "West Bank and Gaza Strip" OR Yemen OR Zambia OR Zimbabwe)) OR  (TITLE-ABS-KEY ("low income" OR "middle income") OR ("low AND middle"[Title-Abstract]) AND countr*))) |
| **#3** | **#1 and #2 AND #3** |

**Google Scholar**

“Co-creation” AND “LMIC” AND “public health”

**Patient-Centered Outcomes Research Institute (PCORI)**

“Co-creation”

**Participedia**“Co-creation” AND “public health”
