# Supplementary material for: Conducting co-creation for public health in low and middle-income countries: a systematic review and key informant perspectives on implementation barriers and facilitators
Source: Global Health. 2024 Jan 17;20:9. doi: 10.1186/s12992-024-01014-2 (PMC10795424; doi:10.1186/s12992-024-01014-2)
Supplement: Supplementary file 4 — Supplementary Material 4: Guiding interview questions [file 12992_2024_1014_MOESM4_ESM.docx]

**Data extraction template**

| **Field** | **Notes** |
| --- | --- |
| Author | [Names of authors] |
| Year | [Year published] |
| Title | [Publication title] |
| Country | [Country in which the study was conducted] (If multiple countries for data collection, then list all here) |
| Public health issue | [Public health issue that the study was targeted to address] |
| Aim | [Main intervention’s aim] |
| Co-creators | [Specify who joined the process, e.g. whether it is a end-user representative or representative of the target population or a community members or a stakeholder, etc.] |
| Approach adopted | [Specify which approach are the authors reporting to be using, e.g. co-creation, co-production, co-design, etc.] |
| Adaptation | [Note whether the solution has been a) developed directly in the LMIC setting or b) originally developed in a HIC setting and then transferred/adapted to a LMIC setting or c) originally developed in another LMIC setting and then transferred/adapted to the study's LMIC setting] |
| Implementation facilitators | [Include all information around implementation enablers, i.e. defined as factors which have increased and improved implementation.] |
| Implementation barriers | [Include all information around implementation barriers, i.e. defined as factors that obstruct the capacity to implement interventions] |
